# 3D Printed Shape Memory Polymers Produced via Direct Pellet Extrusion †

**DOI:** 10.3390/mi12010087

**Published:** 2021-01-15

**Authors:** Trenton Cersoli, Alexis Cresanto, Callan Herberger, Eric MacDonald, Pedro Cortes

**Affiliations:** 1 Civil/Environmental & Chemical Engineering, Rayen School of Engineering, Youngstown State University, Youngstown, OH 44555, USA; tmcersoli@student.ysu.edu (T.C.); accresanto@student.ysu.edu (A.C.); 2 Electrical Engineering, Rayen School of Engineering, Youngstown State University, Youngstown, OH 44555, USA; caherberger@miners.utep.edu (C.H.); emac@utep.edu (E.M.)

**Keywords:** 3D printing shape memory polymers, actuation, counterfeit resistance, morphing structures

## Abstract

Shape memory polymers (SMPs) are materials capable of changing their structural configuration from a fixed shape to a temporary shape, and vice versa when subjected to a thermal stimulus. The present work has investigated the 3D printing process of a shape memory polymer (SMP)-based polyurethane using a material extrusion technology. Here, SMP pellets were fed into a printing unit, and actuating coupons were manufactured. In contrast to the conventional film-casting manufacturing processes of SMPs, the use of 3D printing allows the production of complex parts for smart electronics and morphing structures. In the present work, the memory performance of the actuating structure was investigated, and their fundamental recovery and mechanical properties were characterized. The preliminary results show that the assembled structures were able to recover their original conformation following a thermal input. The printed parts were also stamped with a QR code on the surface to include an unclonable pattern for addressing counterfeit features. The stamped coupons were subjected to a deformation-recovery shape process, and it was observed that the QR code was recognized after the parts returned to their original shape. The combination of shape memory effect with authentication features allows for a new dimension of counterfeit thwarting. The 3D-printed SMP parts in this work were also combined with shape memory alloys to create a smart actuator to act as a two-way switch to control data collection of a microcontroller.

## 1. Introduction

Additive manufacturing, commonly known as 3D printing, comprises many different technologies and processes utilized to manufacture custom parts with a range of materials such as metals, polymers, ceramics, and sand. While the original implementation of additive manufacturing was to yield rapid prototyping parts, 3D printing is now being used to manufacture parts for structural applications including maintenance and repair of damaged structures [1]. Arguably, the most accessible form of 3D printing is the fused filament fabrication (FFF) due to its low cost, suitability for use in an office environment, and availability of a wide range of printing materials [2]. FFF additive manufacturing is a type of material extrusion process in which a thermoplastic filament is heated to its melting point and extruded onto a build platform, creating a layer-by-layer part. A recent extension of this technology is the process of printing with raw pellets instead of a thermoplastic filament. In this process, a polymer extruder is mounted to the printer and raw pellets are fed, heated, and extruded from the print head [3,4]. This process allows for faster printing, larger manufactured parts, and less thermal processing of the polymer in the printed structures [4].

Combining additive manufacturing with smart materials is commonly referred to as 4D printing, introduced by Tibbits in 2013 [5]. Smart materials allow the manufacturing of parts that self-repair, generate a physical response, or create self-assembling structures [6]. 4D printing combines the use of a 3D object with the fourth dimension, often having a second geometry programmed into the part. When the object is subjected to heat [7,8], a change in pH [9,10] water [11,12], magnetic field [13,14] or other external stimulus, the object responds by changing its shape. Combining smart materials with additive manufacturing allows for the production of complex, custom parts with tailored smart-morphing properties.

One smart material of specific interest is the use of shape memory polymers in 3D printing. Shape memory polymers (SMP/s) respond to a stimulus and can be deformed and then return to a permanent or “programmed” shape [15]. Many shape memory polymers are available that can be triggered with different stimuli; for this work, thermally induced shape memory polymers will be considered. Thermally induced shape memory polymers often utilize the glass transition temperature (Tg) of the material to switch between a temporary deformed shape and the set-permanent shape. Above the Tg, the polymer becomes soft and rubbery, allowing it to easily be deformed, and the deformed shape can be solidified by allowing the part to cool below the Tg under deformation. Thermally induced shape memory polymers consist of three distinct phases that allow for the shape memory effect to be observed: The polymer, netpoint domains for the permanent shape, and netpoint domains for the temporary shape [16]. Consequently, shape memory polymers can be characterized based on these netpoints, which can be formed via chemical crosslinking, hard domains associated with a melting temperature (thermoplastics), or hard domains associated with a glass transition temperature (thermosets) [16].

Previous work in additively manufactured shape memory polymers has included multiple technologies for polymer additive manufacturing including: Fused filament fabrication [17,18,19], stereolithography [14,20,21], and digital light processing [22]. While vat polymerization techniques such as stereolithography and digital light processing utilize prepolymers in a liquid state and a light source to cure individual layers of a solid object, fused filament fabrication turns polymer pellets into a cylindrical filament that is then remelted and deposited in a layer-by-layer fashion to form a 3D object. Several works have explored the use of FFF to produce smart materials [19,23].

Of the most relevance to this work is the 3D printing of shape memory polymers based on polyurethane thermoplastics. Villacres et al. [19] processed polyurethane shape memory pellets (SMP technologies) into a filament and fed this material into a FFF 3D printer to study the mechanical properties. Similar techniques of first processing SMP pellets into a filament, and then feeding the filament into a 3D printer have been examined by other researchers [17,24]. While fabricating samples directly from a feedstock material in pellet form has yet to be examined.

As mentioned, the fabrication of thermo-responsive smart materials has many applications. Of specific interest is the incorporation of thermo-responsive materials as actuators in electronic circuits. Zarek et al. [14] examined shape memory polymers produced via Stereolithography (SLA) printing to create a circuit illuminating a light. After the part was manufactured, silver was deposited across the polymer to create electronic contacts, and the SMP was subsequently deformed [14]. Upon heating, the SMP returned to its initial state and the circuit was completed, illuminating the led. It is important to note that this type of actuator must be manually reset upon each use. In else, the circuit would turn on once a stimulus was applied but must be manually deformed to turn the stimulus off. This is due to the typical one-way shape memory effect present on shape memory polymers. Additional research has concentrated on investigating two or multiple shape memory effects on SMPs [25,26,27].

This work focuses on combining the benefits of material extrusion through a direct pellet extrusion process, with advanced materials, to produce smart 3D-printed parts, exhibiting the shape memory effect. To circumvent the manual resetting of an SMP actuated switch, this work solves this problem by preparation of a hybrid part containing both shape memory alloy (SMA) and SMP for use in an electronic circuit. This creates a reversible actuated switch, capable of turning on and off with an external stimulus of heat. In addition, all SMP parts fabricated in this work are created directly from the feedstock material, without the need of first turning SMP pellets into a 3D printing filament. The parts used for analysis and characterization, are 3D printed using the above-mentioned technique of direct pellet extrusion.

## 2. Materials and Methods

### 2.1. Materials and Printing Process

**Figure 1:**
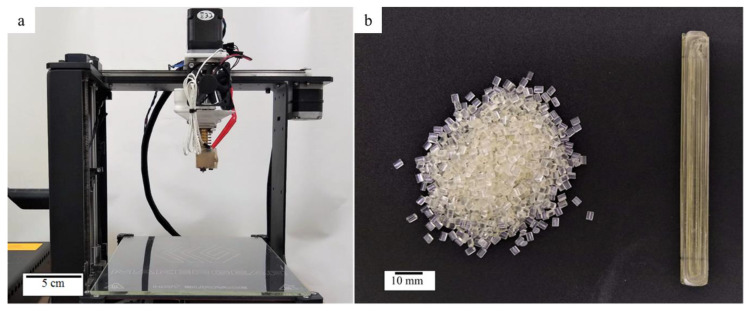
Instrumentation and supplies used in this work. (**a**) Universal pellet extruder mounted on the open-source Makergear M2 Printer. (**b**) Shape memory polymer (SMP) pellets used in this work (**left**) and 3D-printed SMP part (**right**).

In this work, shape memory polymer pellets (Diaplex 9020), designed for use in injection molding applications, were purchased from SMP Technologies incorporated (Tokyo, Japan) and the pellets were used as received, and fed directly into the printer. From the supplier, Diaplex 9020 is a polyurethane-based shape memory polymer, with a glass transition temperature of 90 °C, which can be utilized as the switching temperature to trigger the shape memory effect [28]. The pellets were extruded using a universal pellet extruder (Mahor XYZ), which was then mounted on an open source, fused filament fabrication (FFF) printer (Makergear Beachwood, OH, USA) in place of the printer’s normal extruder (Figure 1).

Prior to manufacturing of 3D parts on the printer, the process was similar to that of common FFF 3D printing. Solid models of the samples were rendered using the software Fusion 360 (Autodesk Inc., San Rafael, CA, USA), or downloaded from the open-source website Thingiverse (Makerbot Industries, Brooklyn, NY, USA). Once the stereolithography (.stl) mesh files were prepared, they were then imported into the open-source software Slic3r in order to generate .gcode files for the printer. Print settings, described below, were selected and modified in Slic3r, and the .gcode file was then loaded to the printer.

The universal pellet extruder utilized a stepper motor to drive an auger that forced the pellets (Figure 1b) through a 1.5 mm nozzle. The samples printed in this work (Figure 1b) were printed at a speed of 5 mm/s, with a layer height of 0.9 mm. Additionally, the nozzle temperature was set at 225 °C, and the bed was set at 80 °C, to assist with adhesion of the print to the build surface.

All other components of the printer remained as built from the manufacturer. The stepper motor for the pellet extruder was connected to the printer’s circuit board. Also, the firmware was modified to provide the correct current to the motor, as it had different requirements than the original extruder motor.

### 2.2. Mechanical Testing

Mechanical tests were conducted to determine the properties of the printed parts. Four tensile samples were tested based on the ASTM D638 (type IV) in an Instron 5967 unit with a load cell of 10 kN. Samples were pulled at a rate of 2 mm/min, and the corresponding stress and strain was collected by the instrument. Flexural testing (ASTM D790) was also conducted, and six rectangular samples (100 × 10 × 4 mm) were printed for examination under compression in a three-point bend flexural test. For both mechanical tests, an average was collected from the samples and the standard deviation was determined.

### 2.3. Thermal Analysis

Differential scanning calorimetry (DSC) was utilized to examine the glass transition temperature and thermal properties of the printed shape memory polymers. This test was conducted using a PerkinElmer hyper-DSC (Waltham, MA, USA) in a range from 25 °C to 230 °C. The printed and virgin pellet material samples (5–10 mg) were prepared in an aluminum pan with a lid. The samples were heated and cooled at a rate of 10 °C/min to 230 °C, and cooled to 25 °C, once to erase the thermal history of the material, and a second time for measurement of the glass transition temperature. Here, the average of three separate samples was used in reporting the Tg of the material. This temperature range was selected based on the Tg stated by the manufacturer of the material (90 °C), and up to a temperature of 230 °C to evaluate any thermal events that may occur during the printing process of the samples.

### 2.4. Shape Memory Testing

**Figure 2:**
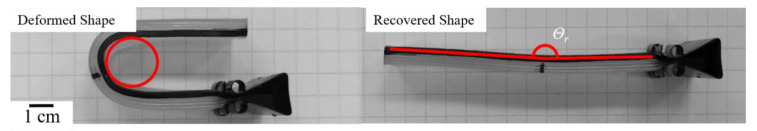
SMP samples for the fold-deploy test. Deformed sample (**left**) and recovered sample showing the recovered angle, *θ_r_*, (**right**). The black line on the sample was drawn on the coupons as the angle reference for measurement.

To examine the thermo-responsive memory effect and determine the shape recovery ratio of the 3D-printed shape memory polymers, the fold-deploy test developed by Liu et al. [29] was utilized. This test quantifies the shape recovery angle of a deformed part after heating and has been used by several other researchers to characterize the shape memory properties of SMPs [30,31,32]. In this test, a flat rectangular specimen (80 mm × 11 mm × 4 mm) was bent into a “U” shape around a rod with a diameter of 8 mm (Figure 2). The manufactured shape is set as the reference angle (*θ_max_* = 180°), and then reheated above the Tg of the material in boiling water. After the shape has returned to its recovered shape and subsequently cooled, the angle (*θ_r_*) is recorded. The shape recovery ratio is then calculated using the following formula:

(1) $$\mathrm{Shape}\;\mathrm{recovery}\;\mathrm{ratio}:\;\%R_{r}=\left(1-\frac{\theta_{max}-\theta_{r}}{\theta_{max}}\right)\times 100\%$$

To determine the shape retention ratio of the manufactured samples, thermomechanical shape recovery cycles were conducted using a TMA Q400 (TA instruments, New Castle, DE, USA). In this test, a small sample of SMP (5 × 10 × 4 mm) was machined from a printed SMP part. The TMA Q400 was operated with the provided macro-compression probe (diameter = 6.07 mm). The steps for the thermal mechanical shape recovery followed closely the procedure from other researchers [33,34,35]. The method of the thermomechanical analysis consisted of equilibrating the sample at 110 °C for 5 min, ramping the applied force (0.5 N/min) to 0.9 N, and setting this deformation by cooling the sample to 25 °C at 3 °C/min. The recovery to the permanent shape is achieved by removing the applied force to 0.001N (at 0.5 N/min), holding the sample at 25 °C for 5 min, and heating the sample back to 110 °C at 10 °C/min and finally holding for 10 min.

The shape retention, or shape fixity ratio, *R_f_*, as defined by Zhao et al. [7] can then be calculated from Equation (2):

(2) $$\%R_{f}=\frac{\varepsilon_{applied}}{\varepsilon }\;\times \;100\%$$

where *ε_applied_* is the strain from the applied load of 0.9 N, and *ε* is the strain remaining in the sample after the load is removed (to 0.001 N). This property demonstrates how capable the shape memory polymer is of retaining the deformed state.

### 2.5. Authentication

The ability of incorporating a unique identifier into the manufactured parts was examined in this work, within the context of intentionally hiding a fingerprint using the shape memory effect. Previous work by Pretsch et al. [36] has shown the application of quick response (QR) codes printed on shape memory polymers. The combination of the materials shape memory effect allows the QR code to be distorted and unscannable when the part is deformed, and scannable once the part has returned to its original shape. Challisery et al. [37] demonstrated this by 3D printing a QR code on a shape memory polymer material. In this work, the applicability of this authentication technique is addressed on the printed Diaplex 9020 SMP. The SMP coupon was placed in a Roland VeraUV substrate printer (Irvine, CA, USA) to create the QR-code design onto the part. The Logojet deposited a UV-curable ink onto the 3D part in a pre-generated design, which consisted of a working QR code surrounded by unscannable QR-codes, to disguise the exact location. Lastly, the QR Code was verified to work by scanning with a smartphone, before undergoing thermal deformation and the shape memory return.

### 2.6. Actuation of a Microcontroller with a 3D Printed Smart Switch

**Figure 3:**
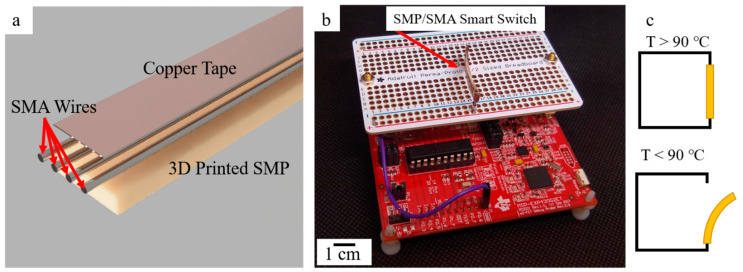
(**a**) Digital render (Fusion 360) showcasing the design of the SMP/shape memory alloy (SMA) smart switch. (**b**) The fabricated SMP/SMA switch connected to a breadboard and the MSP430. (**c**) A diagram illustrating the switch in the “on” position (**top**), and “off” position (**bottom**).

To demonstrate potential applications of additively manufactured shape memory polymers, a printed sample (28 × 5 × 1 mm) was combined with a shape memory alloy (SMA) wire to act as a counter-balanced switch in a simple electric circuit (Figure 3). Here, nickel-titanium wires (Dynalloy, Irvine, CA, USA), with a transition temperature of 90 °C, were glued to the printed SMP part, and a piece of copper tape was affixed to allow current to pass through the smart switch. The SMP/SMA hybrid part was then used as a thermal switch in a circuit. Once heated, the SMA wire straightened out, causing the entire part to straighten. Upon removal of the heat, the switch relaxed into a slightly curved geometry. The smart sensory switch was here used to actuate a microcontroller. The microcontroller was a Texas Instruments (Dallas, TX, USA) MSP430 G2553 (see Figure 3), and serial data were collected through the MSP430 development board. The temperature sensor present in the G2553 microcontroller was utilized to collect temperature data. Here, the entire board and smart switch was set into a laboratory oven, when the switch reached the transition temperature, the microcontroller was triggered to initiate temperature data collection. When the smart switch returned to the “off” state, the process was stopped. Several pins of the G2553 were required for the data transmission and the powering of the microcontroller. One pin was set as an input and it was this pin that would trigger the collection of data through an interrupt. The data were transferred from the G2553 through UART.

In this example of a smart switch to collect temperature data, all temperature information above 90 °C were collected. The continuous collection of data by the MSP430 and smart switch system was achieved at temperatures above 90 °C. As mentioned previously, the system was placed in a laboratory oven (at room temperature) subjected to a temperature ramp to 95 °C. After 5 min, the oven temperature was then increased to 105 °C and held for an additional 5 min. The oven was then switched off, and cooled passively for 30 min, until the oven door was opened. Once opened, the system cooled rapidly, and the smart switch then disconnected from the board, and data collection stopped.

## 3. Results and Discussion

### 3.1. Mechanical Properties

**Figure 4:**
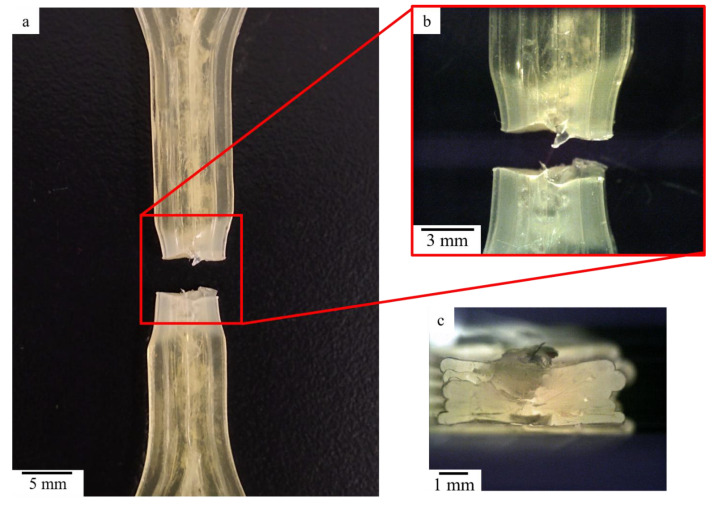
Optical microscope images taken of (**a**) fracture area of tested tensile specimen; (**b**) magnified view of fractured area; (**c**) cross-section of fractured area.

The average tensile strength of the printed samples was 55.58 MPa, with a standard deviation of 2.29 MPa. It should be noted that the manufacturer reported the tensile strength of the material to be 48 MPa; however, measurements of higher ultimate tensile strength (UTS) than that reported from the manufacturer has also been reported by Villacres et al. [19] on an SMP material (MM4520). The samples prepared in this work were printed with g-code settings for 100% infill. Given that the samples consist of longitudinal layer lines and yielded higher UTS than the injection molded counterparts due to alignment of the polymeric chains, it is important to note that the layers of the tested samples (Figure 4) were printed and tested in the longitudinal direction. Figure 3 shows a clear necking on the samples following the tensile testing, a mechanism that suggests a certain degree of ductility. Included in Figure 4 is the micrograph of the cross-sectional area of the tested sample, where a limited level of porosity is observed, indicating a high degree of densification during the printing process.

Examination of the flexural properties showed that the printed samples exhibit a flexural stress of 73.4 MPa, with a standard deviation of 3.44. Here, it is important to note the tested specimens did not fracture during the three-point bend test, instead they remained intact. Once again, the examined flexural strength (73.4 MPa) is higher than the manufacturer’s reported bending strength of 55 MPa.

### 3.2. Differential Scanning Calorimetry

**Figure 5:**
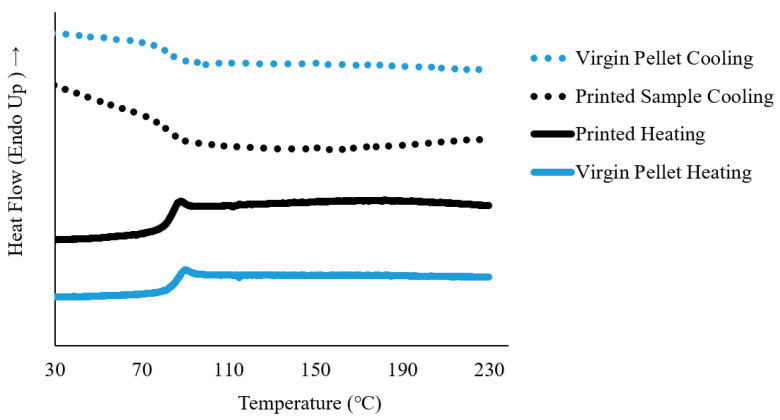
DSC Heat flow thermogram of the second heating and cooling cycle of the virgin pellet, and printed Diaplex 9020 sample.

Differential scanning calorimetry (DSC) exposed the transformation temperature of the printed SMP Samples. The results from the DSC are shown in Figure 5, for both the virgin pellet and unprinted material. From the second heating ramp, it was determined that the glass transition temperature of the printed material occurred at 85.4 °C (±0.74 °C), which is slightly lower than the reported from the manufacturer (90 °C). In the cooling curve of the printed sample, the glass transition temperature was observed at 81.9 °C (±1.15 °C). While this measured result is slightly lower than the manufacturer’s reported data, it should be noted that the manufacturer reports the glass transition as an inflection of the modulus of the material. Since the measured value by DSC and the reported value is within 5 °C, this difference can be attributed to the difference in techniques of measuring the glass transition temperature [38]. No additional peaks were present in the thermograms of the printed and virgin material, indicative of the amorphous nature of this polyurethane shape memory polymer network.

### 3.3. Shape Memory & Authentication

**Figure 6:**
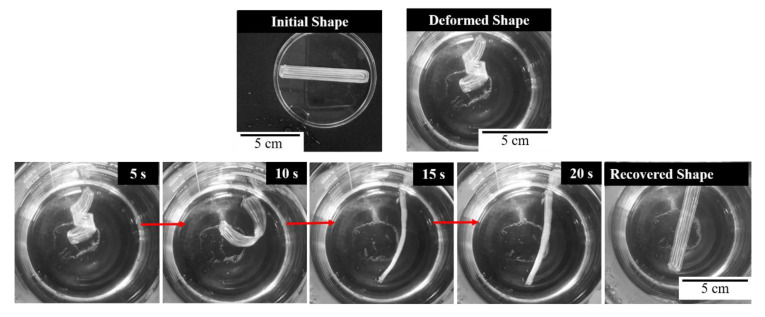
The shape recovery of a flat bar in hot water.

The shape memory effect was first tested in water at 95 °C. A rectangular bar was 3D printed and deformed by hand at elevated temperature. From the photos in Figure 6, the recovery of the shape from a twisted shape to a flat bar is observed. Here, the twists applied in the deformed shape are unraveled as the SMP recovers its permanent shape. The recovered shape is achieved in under 25 s of free recovery in hot water.

**Figure 7:**
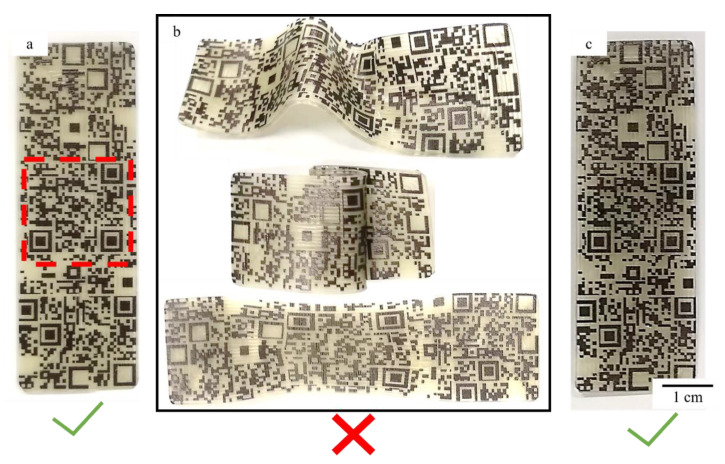
(**a**) SMP printed shape with printed QR-code (outlined in the red dashed square). (**b**) Samples after heating and deformation. (**c**) Recovery of the sample after heating.

The presence of the shape memory effect in the printed specimens was then employed in the aforementioned authentication method. The inclusion of this counterfeit mechanism is due to the increased need of finger printing features in smart microelectronics. In this work, a simple QR code image has been incorporated into the printed SMP. Here, the specimen was scanned with a smartphone revealing the disguised QR code. Next, the sample was heated with a heat gun and deformed, resulting in the QR code being undetectable. Finally, the entire sample was heated again, and the QR code was scanned again with a smartphone. Pictures of the sample throughout this test can be seen in Figure 7. This test confirmed the applicability of QR codes as an undetectable authenticator for shape memory polymers.

### 3.4. Shape Recovery Cycle

**Figure 8:**
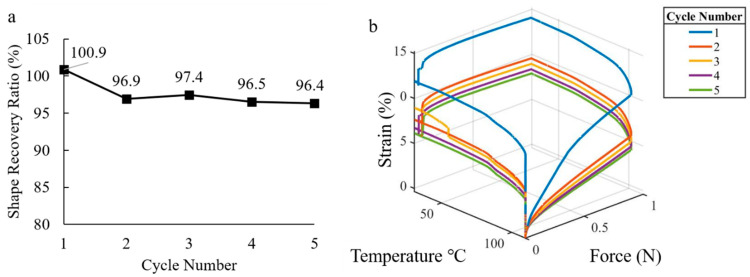
Thermal cycle of Shape memory polymer vs. shape recovery ratio (**a**), strain % and load (N) as function of temperature (**b**).

The results of the fold-deploy test are shown in Figure 8. Here it was observed that the first deformation had a recovery of 100.9%, and subsequent deformations approached a recovery of 96.4%. The fact that the shape memory polymer recovered more than the initial shape (100.9%) could be associated to the presence of residual strains induced during the printing process. These residual strains appear to be released after the first thermal cycle. A similar behavior is shown from the plot of temperature, strain %, and load (Figure 8) where it can be seen, while the load was constant for each cycle (0 to 0.9 N), the first cycle resulted in a higher deformation of the sample. This result is consistent with other thermomechanical tests of shape memory polymers [35,39,40]. While the shape recovery decreased and converged to a value with subsequent thermal cycles, the shape retention ratio remained constant with each thermal cycle. From the thermomechanical shape recovery test, the shape retention ratio did not significantly decrease with different cycles and measured 90.7% with a standard deviation of 0.9%. Together, the fold-deploy test and thermomechanical shape recovery analysis illustrate that the 3D printed SMP samples largely exhibit the shape memory effect.

### 3.5. Application of the SMP as a Thermal Switch

**Figure 9:**
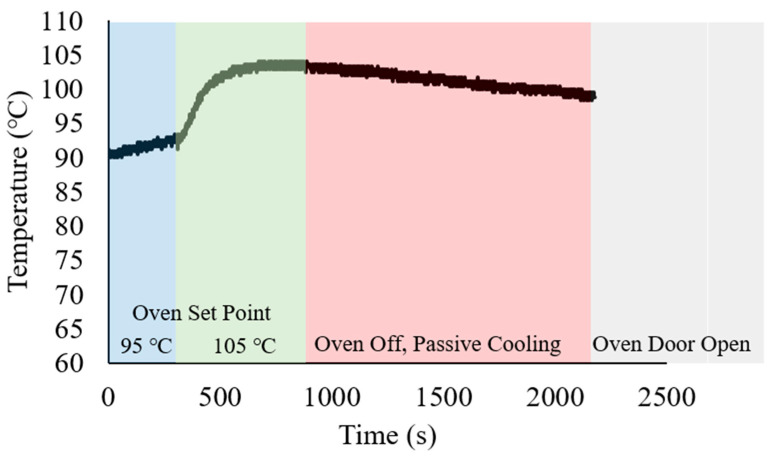
Data collected from the MSP430 controlled by the SMP/SMA switch. Temperature set points of the oven were changed with time, and temperature information above 90 °C was collected.

To illustrate the actuation of data collection via the MSP430 G2553 microcontroller, the hybrid SMP/SMA switch was mounted to a circuit board. This switch takes advantage of the distinct rubbery and glassy phase of the printed SMP material and mechanical behavior of the SMA. The actuator consisting of the SMP printed switch, shape memory alloy wires, and copper tape behaved as such: Upon the thermal stimulation (T > 90 °C) the SMP became rubbery, and the SMA wires straightened. Once straight, the switch connected to a pin on the breadboard, signaling the microcontroller to begin the data collection. By design, once the temperature dropped below 90 °C, the SMA loses its actuation force and the SMP recovers the bending shape, making the switch disconnect from the pin, and ending the data collection. Dynamic data collection from the smart switch was achieved, resulting in Figure 9, the temperature reading from the smart sensory printed circuit. Here, it was observed the data recording from 90 °C, showing the oven heating to 105 °C, a decrease in temperature as the oven cools, to an abrupt cooling in the system after 40 min. Similar readings were observed in several trials on the system. The manufactured sensor clearly shows a practical application of smart electronics using a 3D printing technology.

## 4. Conclusions

Through the process of direct pellet extrusion, an open-source FFF 3D printer has been modified to 3D shape memory polymer parts. Using commercially available SMP pellets, smart coupons were manufactured having an ultimate tensile strength of 55.58 MPa, with a flexural strength of 73.4 MPa. The 3D-printed parts had a measured glass transition temperature of 85.4 °C, which acted as the transformation temperature of the SMP. The 3D-printed shape memory polymer parts exhibited a shape recovery over 96% and a shape retention of 90.7% across the investigated thermal cycles. The thermal triggering of shape recovery and deformation of the 3D-printed parts were achievable by both hot water and the use of a heat gun.

Finally, the 3D-printed smart materials were observed for their applicability in enhanced authentication and electronic structures. The usage of printed SMP parts was successful in making a QR code that is undetectable when deformed, and detectable when a thermal stimulus is applied. SMP and shape memory alloy parts were combined into a hybrid actuator that successfully functioned as a thermal switch. Through a change in temperature, the switching component deformed and relaxed to two distinct phases, allowing the actuation of an electronic circuit.

## Data Availability

Data available upon request from the corresponding author.
